# Addendum: Amplification, Sequencing and Cloning of Iranian Native *Bacillus subtilis* Alpha-amylase Gene in *Saccharomyces cerevisiae* [published in Jundishapur J Microbiol. 2013;6(8):e7371]

**DOI:** 10.5812/jjm.17783

**Published:** 2014-02-01

**Authors:** Fahimeh Afzal-Javan, Mohsen Mobini-Dehkordi, Behnaz Saffar

**Affiliations:** 1 Department of Genetics, Faculty of Science, University of Shahrekord, Shahrekord, IR Iran; 2 Research Institute of Biotechnology, University of Shahrekord, Shahrekord, IR Iran

**Keywords:** Industrial Enzyme, Cloning, Alpha-amylase

## Addendum

This addendum supplies corrected statements and proofs of an error by author. Hear by, authors declare that The correct authors list and affiliations are:

Fahimeh Afzal-Javan ^1,2^, Mohsen Mobini-Dehkordi ^1,2,*^, Behnaz Saffar ^1,2^

^1^ Department of Genetics, Faculty of Science, University of Shahrekord, Shahrekord, IR Iran

^2^ Research Institute of Biotechnology, University of Shahrekord, Shahrekord, IR Iran

Mohsen Mobini-Dehkordi

